# UAV-WPT System Based on Novel Magnetic Structure and Model Predictive Control

**DOI:** 10.3390/s23156859

**Published:** 2023-08-01

**Authors:** Qi Wang, Xingcan Li, Fei Yang, Tian Gao

**Affiliations:** 1 School of Electronic Information Engineering, Xi’an Technological University, Xi’an 710021, China; wangqi@xatu.edu.cn (Q.W.); lixingcan97@163.com (X.L.); yangfei@st.xatu.edu.cn (F.Y.); 2 School of Electronic and Information, Northwestern Polytechnical University, Xi’an 710072, China

**Keywords:** UAV-WPT, orthogonal coupling structure offset, offset weight method, model prediction control

## Abstract

In this paper, a UAV-WPT based on a new orthogonal coupling structure is first designed. An offset weight method is proposed to optimize the design of the system resonance parameters to improve the power transmission efficiency in the offset state. The coupling mechanism’s coupling ability and anti-offset performance are experimented with using Ansys software simulation. The system adopts LCC-S topology and proposes a secondary side constant current control strategy based on the model prediction control to ensure constant current output in the case of coupling coefficient deviation. Finally, an experimental platform is designed for experiments. The results show that the system can quickly realize 3A constant current output under the change of load and coupling coefficient, and at the same time, improve the working efficiency of the system.

## 1. Introduction

Unmanned aerial vehicles (UAVs) have the advantages of flexible control, simple structure, and low cost, and are widely used in monitoring, plant protection, inspection, disaster relief, etc. [1,2,3]. The flight time of a UAV is an important indicator to measure its performance, and it is a key factor in determining the flight range, the amount of information obtained, and the quantity and quality of tasks performed by a UAV. Therefore, how to effectively extend the flight time of UAVs is a key issue restricting the development of UAVs in the context of no significant improvement in battery energy storage capacity.

There are two ways to prolong the flight time of drones. The first is to increase the capacity of lithium batteries, but the load capacity of drones is limited, and overweight lithium batteries will affect their performance. If the traditional wired plug-in charging method is used, it will consume a lot of manpower and reduce the flexibility of the drone. If the charging process does not require manual intervention, the efficiency of the drone’s mission will be greatly improved. Paper [4] proposes that photovoltaic cells can be installed on the wings of UAVs, but this method is too dependent on solar radiation, which greatly limits the working time of UAVs. Paper [5] proposes laser beam technology to charge UAVs, but the power source of this method must always be kept close to the UAV, which increases the cost of UAV operation, and the laser will cause serious harm to organisms.

Wireless power transmission (WPT) can realize the wireless transmission of energy through non-physical direct contact. It has the advantages of high security, strong reliability, and easy automation of the charging process. Therefore, wireless power transmission technology can be applied to the field of drones. Establishing an unattended charging base station wirelessly charges the UAV to make up for the limitation of its short-term operation. At present, there are a few types of research on the wireless power transmission technology of drones. In paper [6], a small receiving coil is installed at the bottom of the landing gear of the UAV. The magnetic field of this coupling device has a small space and has little impact on the equipment of the UAV. However, the small receiving coil has a poor ability to capture magnetic flux and cannot perform high-power transmission. Paper [7] installed a hollow planar, receiving coil on the abdomen of the UAV, so that the coil area of the fuselage was significantly smaller than the area of the transmitting end. However, in this coupling device, since the receiving coil of the system is directly installed on the abdomen of the UAV, it occupies the installation space of the UAV’s pan/tilt, which seriously affects the operation performance of the UAV. Paper [8] developed a 51 W six-wing UAV wireless power transmission system with a maximum charging efficiency of 63.4%. And the external environment will easily make the six-wing UAV unbalanced and difficult to control. Paper [9] designed a conical convex structure for the wireless charging of drones and the designed coupling device structure. In the process of UAV docking, by relying on the constraints of the physical shape of the coupling device, the UAV can directly stop at the designated transmission position, but this solution needs to modify the UAV style, and increases the design cost of the coupling device. Paper [10] used the method of expanding the transmitting coil to realize WPT but did not optimize the parameters of the coupling mechanism, and the transmission efficiency was only 62%.

The WPT system for small UAVs is easily affected by various factors such as the environment during the working process. This will directly affect the stability of the UAV during the charging process and may even cause the system to stop working. In the wireless power transmission control system, the control strategy can be divided into primary side control and secondary side control according to the location of the control [10]. Primary side control refers to controlling the output current by adjusting the parameters of the power input terminal [11]. This method can generally be divided into two methods: adjusting the excitation current of the transmitter and adjusting the operating frequency of the transmitter. However, in actual use, it is often necessary to add an auxiliary wireless communication module. Otherwise, it is necessary to estimate the transmission state of the system by collecting the voltage and current information of the primary side, and the control accuracy of the system depends entirely on the accuracy of the estimation algorithm. When the estimation accuracy of the estimation algorithm is low, the effect of the primary side control is not ideal. Secondary side control is a method to realize output current regulation by controlling the receiving end, which can complete the control of system output without wireless communication [12,13]. Paper [14] proposes to place the planar air-core receiving coil under the center plate of the frame, the system transmission power is 70 W, the efficiency is 89%, and the secondary side DC–DC circuit is used to charge the system with a constant current. However, the constant current effect is poor when the mutual inductance fluctuates due to environmental changes after the UAV docks. At present, although some scholars have proposed new methods to control the work of DC–DC circuits, they are not all applied to UAV-WPT systems [15,16,17].

With regard to analyses conducted in previous research, wireless charging technology in the field of UAV-WPT application is not yet mature. The present UAV-WPT system faces several important problems that needs to be solved because of the unique shape, small size, and limited capacity, and so, the design needs to be improved to be able to adapt the system to the drone structure and have a good coupling mechanism. At the same time, due to the changeable working environment of the UAV, the UAV can still charge stably when small-range dislocation, resonance parameter deviation, and load change occur after the UAV lands, which is another important problem faced by the current UAV wireless charging system. In order to solve these problems, the research on UAV-WPT technology mainly focuses on the design scheme of magnetic coupling devices and the transmission performance control strategy.

The coupling mechanism of the primary and secondary sides of the UAV wireless charging system is prone to transverse and longitudinal bias during docking; the system coupling coefficient changes, and the transmission efficiency and output power of the system are significantly reduced. The existing research on system parameter optimization only considers the optimization of system transmission efficiency and output power when the primary- and secondary-side coils are aligned, and the optimization results cannot be obtained under the condition of bidirectional migration.

Aiming at solving the above problems, in this paper, we have designed a reliable and effective wireless charging system for UAVs. Based on the quadratic distribution of the offset of the UAV, an offset weight method is proposed to optimize the resonance parameters, and the coupling mechanism is designed to improve the efficiency of the UAV and the anti-offset ability of the aircraft wireless power transmission system. Secondly, the sub-side control technology is proposed to control the working time of the DC–DC MOS tube on the output side by using a model prediction control algorithm to achieve an accurate constant current for charging UAVs. Finally, we have also designed a WPT system that works at 85 kHz and has an output power of 45 W, and have verified the reliability of the coupling mechanism and control method through experiments.

## 2. Coupling Structure and Parameter Design

### 2.1. Compensation Structure of WPT System

There are four basic compensation networks for wireless charging: primary-side series–secondary-side series connection (SS); primary-side parallel connection–secondary-side series connection (SP); primary-side series connection–secondary-side parallel connection (PS); and primary-side parallel connection–secondary-side parallel connection (PP). The value of the compensation capacitor in the SS and SP compensation methods has nothing to do with the load resistance. However, the value of the compensation capacitance of the PS and PP compensation methods is related to the load resistance [18]. During the charging process, the battery’s equivalent load resistance value will change, the system circuit cannot always be in a resonant state, and a high-efficiency transmission cannot be achieved. Compared with the SP and PS compensation structures, the output of the SS compensation is more stable, but this structure has a problem of lower efficiency.

**Figure 1:**
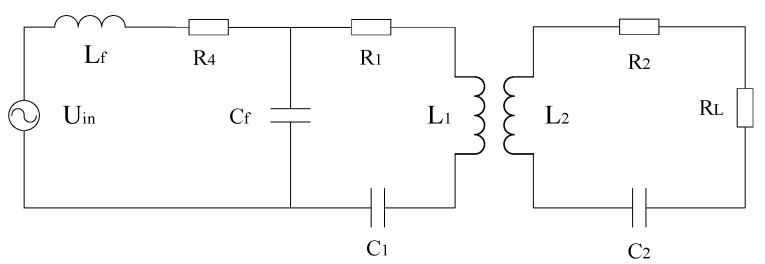
LCC-S-type compensation topology.

In the LCC topology, the transmitter compensation capacitor $C_{f}$ resonates with the self-inductance $L_{1}$ part to reduce the inductance $L_{f}$ value, which will increase the current flowing into the transmitter after inversion. Compared with the SS structure, the transmission efficiency of the system can be improved. Therefore, in this paper, an LCC-S topology that further compensates the primary side power is selected to be used for small UAVs to improve the system efficiency. The topology is shown in Figure 1.

In Figure 1, $U_{in}$ is the input voltage source of the system; the $L_{1}$, $L_{f}$, $C_{f}$ and $C_{1}$ of the transmitting end are the coil self-inductance, compensation inductance, compensation capacitance, and resonance capacitance, respectively; the $L_{2}$ and $C_{2}$ of the receiver are the coil self-sensing and compensation capacitance, respectively; $R_{1}$ and $R_{2}$ are the internal resistance of the transmitting and receiving coils; $R_{L}$ is the equivalent resistance of the rectification link and the load; and $R_{4}$ is the internal resistance of the power supply. When the compensation method is in the resonant state, the following relationship exists:

(1) $$\left\{\begin{array}{c} \omega L_{f}=\frac{1}{\omega C_{f}}\\ \omega L_{2}=\frac{1}{\omega C_{2}}\\ \omega L_{1}=\frac{1}{\omega C_{1}}+\frac{1}{\omega C_{f}} \end{array}\right.$$

The receiving-side circuit impedance is expressed as:

(2) $$Z_{S}=R_{2}+R_{L}+j\omega L_{2}+\frac{1}{j\omega C_{2}}$$

The reflection resistance equivalent to the emission side is:

(3) $$Z_{r}=\frac{\omega^{2}M^{2}}{Z_{S}}$$

The total input impedance at the transmitting end is:

(4) $$Z_{in}=R_{4}+j\omega L_{f}+\frac{1-\omega^{2}L_{1}C_{1}+j\omega C_{1}Z_{r}}{R_{1}-\omega^{2}C_{1}(Z_{r}C_{f}-L_{1}R_{1})+j\omega (C_{f}-\omega^{2}L_{1}C_{1}C_{f}+C_{1}Z_{r}R_{1}+C_{1})}$$

When the system is in resonance, the system output current is [19]:

(5) $$I=\frac{\omega^{2}ML_{f}^{}U_{in}}{(R_{L}+Z_{r})Z_{s}Z_{in}}$$

When the system is in resonance, the transmission efficiency of the system is:

(6) $$\eta =\frac{I^{2}R_{L}}{U_{in}Z_{in}}={\left[\frac{\omega^{2}ML_{f}^{}}{(R_{L}+Z_{r})Z_{s}}\right]}^{2}\frac{R_{L}}{Z_{in}}$$

### 2.2. Parameter Identification of the Battery Model

**Figure 2:**
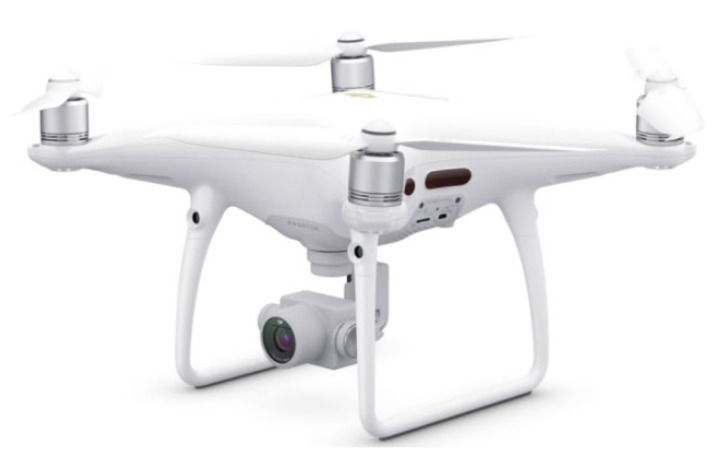
Schematic diagram of a small UAV.

The design of the wireless charging coupling device for small UAVs is not the same as the design of the coupling mechanism for electric vehicles. The schematic diagram of the human–machine structure is shown in Figure 2.

**Figure 3:**
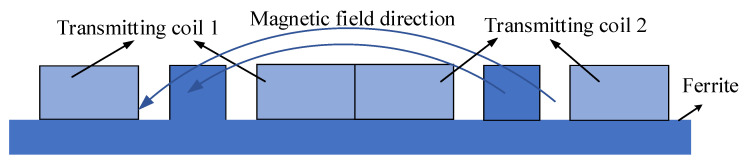
Schematic diagram of magnetic field transmission of orthogonal coupling device.

Therefore, this time, the receiving coil of the drone charging system is wound on one side of the drone’s leg, so that power transmission can be achieved without changing the shape of the drone. This method can not only reduce the wind resistance faced by the UAV during flight, but also better adapt to the shape of the UAV. At this time, the UAV’s wireless charging system will constitute an orthogonal coupling device, as shown in Figure 3.

**Figure 4:**
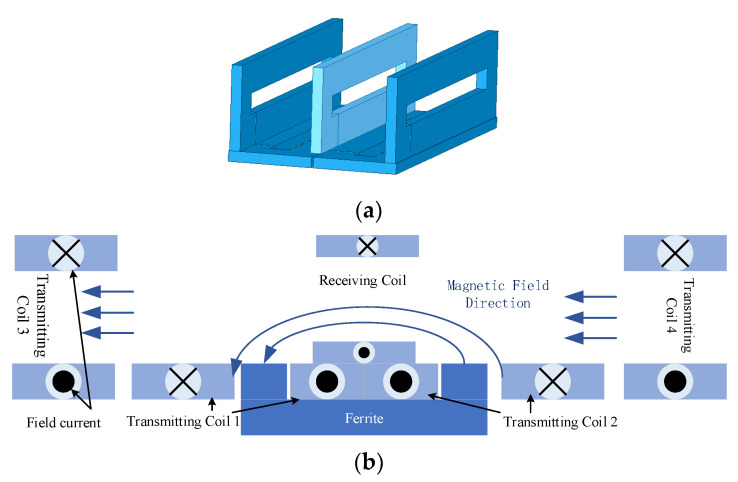
Diagram of the new orthogonal coupling device. (**a**) A new orthogonal coupler model. (**b**) Schematic diagram of magnetic field transmission of new orthogonal coupling device.

To further improve the coupling ability of the bipolar coupling coil, the orthogonal transmitting device is improved, and a new orthogonal coupling device with a pair of vertical rectangular coils will be added on both sides of the receiving coil. When used, the two rectangular coils will generate a magnetic field in the same direction as the orthogonal coil, thereby enhancing the magnetic field strength on the receiving coil, so that the receiving coil can obtain more coupled magnetic fields. The shape and magnetic field distribution of the device is shown in Figure 4.

**Figure 5:**
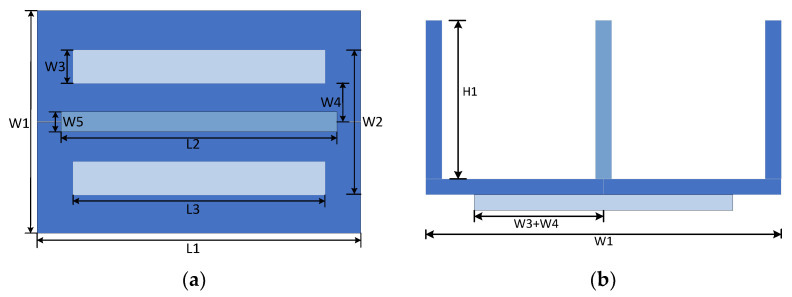
Top and front views of the coupling device. (**a**) The front view. (**b**) The top view.

When designing the size of the new orthogonal coupling device, because the size of the landing gear of the drone is directly determined by the model of the drone, the receiving coil can be directly embedded in the landing gear of the drone. After determining the UAV model, you can directly determine the size of the receiving coil length L2 and receiving coil width W5. The transmit coil width W1 depends on the transmit core width W3 and the transmit coil diameter W4. The top view and front view of the coupling device are shown in Figure 5.

In this design, the length L2 of the receiving coil of the UAV is selected as 120 mm and all coils are made of copper wire. In order to ensure that the size of the transmitting coil can meet the influence of the offset of the UAV, the length of the transmitting coil needs to be slightly larger than the length of the receiving coil of the UAV. The transmitting coil length L1 is set to be 140 mm. Considering that the UAV will be affected by the offset factor when it lands, and considering the position factor of the second rack of the UAV, it is determined that the total width W1 of the UAV’s transmitting coil is 90 mm.

After determining the length and width of the transmitting coil of the new orthogonal coupling device, it is also necessary to determine the coil diameter W4 and receiver coil height H1. In order to ensure the small size, the coil diameter of the receiving coil is consistent with that of the transmitting coil. The established coupling mechanism model is imported into Ansys finite element software. Then, the parameter values of W4 and H1 are scanned by building a finite element model in Ansys. To maximize the coupling coefficient, we set the value of H1 to 45 mm and W4 to 20 mm.

For the landing gear of the small UAV, the parameters of the coupling device designed this time are shown in Table 1.

**Figure 6:**
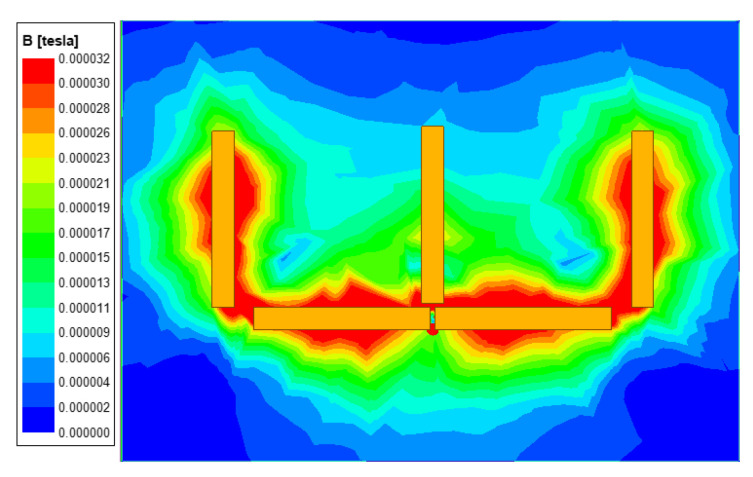
Magnetic field diagram of coupling device in X–Z direction.

The magnetic flux leakage of the system is an important aspect to be considered in the design of the wireless charging system. Ansys Maxwell finite element simulation tool is used to construct the simulation model of the coupling device. The cross-sectional magnetic field distribution is shown in Figure 6. The orange boxes indicates the magnetic field strength. The receiving coil is surrounded by a green uniform magnetic field. It can be found that the transmitting magnetic field is distributed near the transmitting platform, and the receiving coil can capture the transmitting magnetic flux well.

### 2.3. System Resonance Parameter Design

When the shape is completed, the self-inductance parameters of the transmitting and receiving coils of the system can be obtained by measurement, but for the LCC-S compensation structure, the compensation parameters of the remaining coupling mechanism still need to be designed. Considering that the UAV will have the problem of coil offset during the landing process, a parameter optimization method of the UAV coupling mechanism with the goal of anti-offset is proposed in the design of resonance parameters to effectively improve the system performance and anti-offset capability.

#### 2.3.1. Offset Weight Method

**Figure 7:**
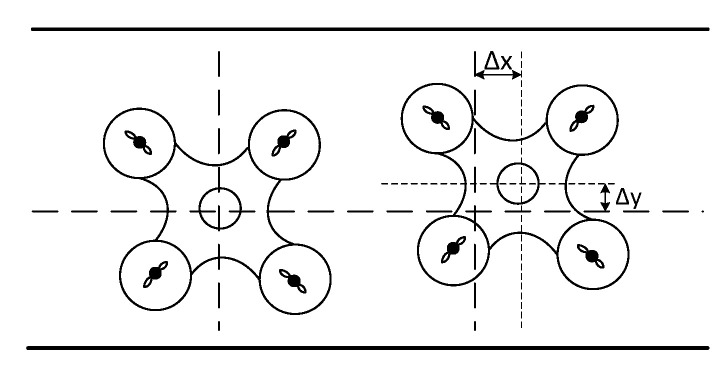
Schematic diagram of unmanned aerial vehicle charging and docking offset.

The actual scene when the UAV is docked is shown in Figure 7. The UAV on the left shows that the UAV is facing the charging coil, and the right side is a schematic diagram of the UAV’s charging offset, where Δx and Δy are, respectively, offset when docked.

To solve the problem of x and y deviation during unmanned class docked, an offset weight method is proposed at this time. This method needs to define the offset weighting coefficient *λ* corresponding to different coupling coefficients. At this time, the weighting efficiency of the system can be obtained as shown in Equation (7):

(7) $$\eta =\lambda_{1}\eta_{1}+\lambda_{2}\eta_{2}+\lambda_{3}\eta_{3}+\cdots +\lambda_{n}\eta_{n}$$

In the equation: $\lambda_{1}+\lambda_{2}+\lambda_{3}+\cdots +\lambda_{n}=1$.

In this weight design, considering the law of drone docking accuracy, we can think that the overall law conforms to the normal distribution when the drone lands, so after the drone lands and docks, it can be considered to obey the normal distribution *N*(0, 0, $\sigma^{2}$, $\sigma^{2}$), so the process follows the 3σ principle, that is, 99.73% of the possible offset values are included within the range of ±3σ.

**Figure 8:**
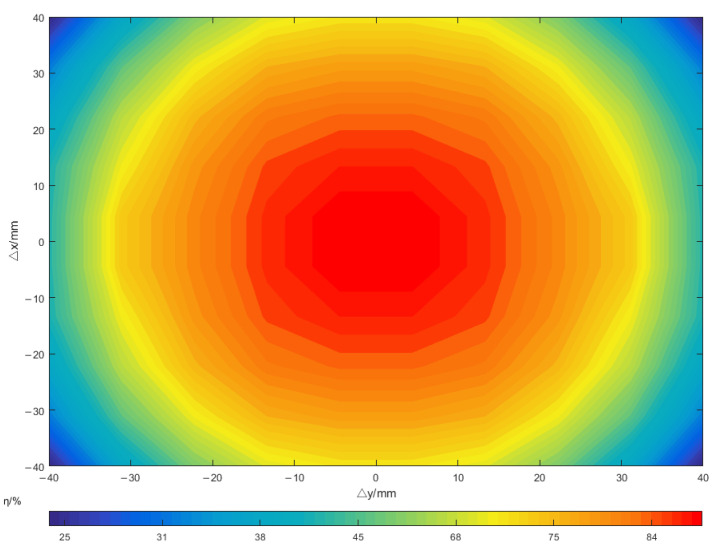
The relationship between the two-dimensional normal distribution and the efficiency change in horizontal and vertical migration.

In the analysis of the size of small drones on the market, the offset range is limited to 60 mm, considering the offsets in the X and Y directions, namely ±30 mm. Figure 8 is the corresponding relationship between the two-dimensional normal distribution obtained in the simulation and the efficiency change of the primary and secondary coils when the horizontal and vertical offsets occur.

Among them, red, yellow, and green represent the states of high, medium, and low efficiency, respectively. It can be seen that the efficiency distribution map obtained by simulation has a significant correspondence with the normal distribution model. The variation of system transmission efficiency with lateral offset distance presents a normal distribution law. According to the simulation results in Figure 7, and through the characteristics of the normal distribution, it is selected when optimizing the parameters. At this time, it meets the range required by the design. Therefore, the probability density at different offset moments can be calculated through the expression of the two-dimensional normal distribution function, and the distribution weight can be obtained on this basis. The probability values under different offsets are:

(8) $$\left\{\begin{array}{l} P(-\sigma \leq \Delta x\&\Delta y\leq \sigma )=57.4\%\\ P(-2\sigma \leq \Delta x\&\Delta y\leq -\sigma U\sigma \leq \Delta x\&\Delta y\leq 2\sigma )=27.9\%\\ P(-3\sigma \leq \Delta x\&\Delta y\leq -2\sigma U2\sigma \leq \Delta x\&\Delta y\leq 3\sigma )=14.4\% \end{array}\right.$$

The corresponding weights under different offsets are allocated by the offset ranges of the three different sums, Δx and Δy, as shown in Table 2.

Through the simulation, it can be obtained that the coupling coefficient of the new orthogonal coupling device designed this time is 0.37. And the weight sequence corresponding to the coupling coefficient in the three-stage design can be obtained by assigning weights through the probability density distribution obtained via the derivation in Table 2 for $\lambda_{i}=\left\{0.57,0.28,0.15\right\}$. The coupling coefficient sequence $k_{i}=\left\{0.35,0.332,0.31\right\}$ is selected in consideration of the offset and is combined with the simulation.

The offset of the system coil is considered when selecting the objective function, and the weighted efficiency is optimized as the goal, so the optimization function can be expressed as:

(9) $$\max\left[\sum_{i=1}^{3}\lambda_{i}\times \eta_{i}\right]$$

(10) $$s.t.\left\{\begin{array}{l} 0\leq p\leq 1\\ 30\leq U_{out}(k=0.2)\leq 50\\ 20\leq U_{out}(k=0.17)\leq 30\\ 10\leq U_{out}(k=0.1)\leq 20 \end{array}\right.$$

Among them, p represents the ratio of the compensation inductance value to the actual inductance value, and $U_{out}$ represents the system output voltage.

#### 2.3.2. Design of Resonance Parameters

**Figure 9:**
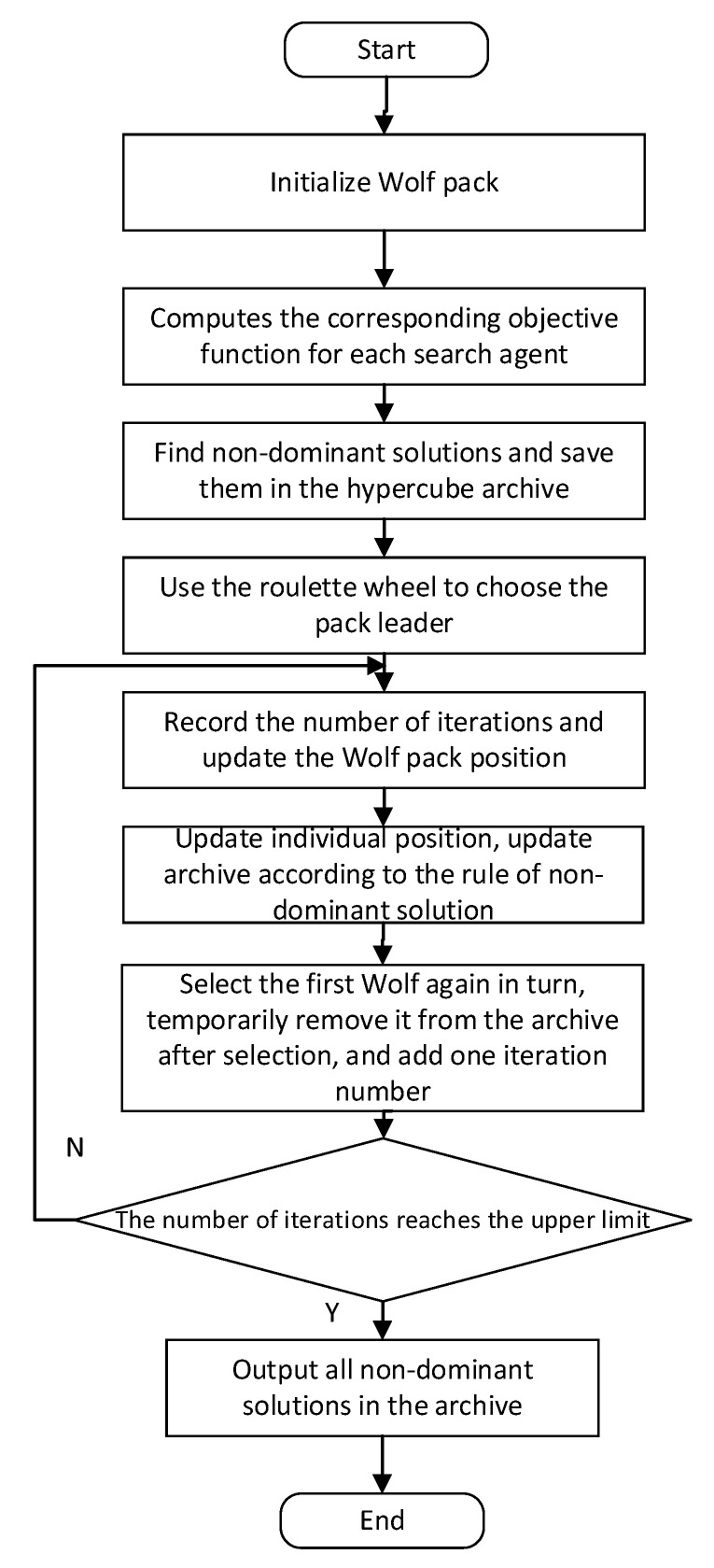
Gray wolf optimization flow chart.

The gray wolf algorithm with good optimization ability is used to solve the above optimization problem [20]. The process is shown in Figure 9. According to the experiment, when the number of iterations is 200, the Pareto surface can reach convergence. Therefore, the number of iterations is set to 200, the size of the wolf pack is set to 100; the control parameter is *a* = 0.1; the swing factor *A* = 10, *C* = 2; and the roulette coefficient is set to 2.

When the number of iterations reaches 30, the fitness function begins to converge, and the weighted transmission efficiency of the system can reach more than 86%. At this time, the proportional coefficient obtained after optimization is p = 0.39, and the parameter values of each component are further calculated through the resonance relationship as shown in Table 3.

**Figure 10:**
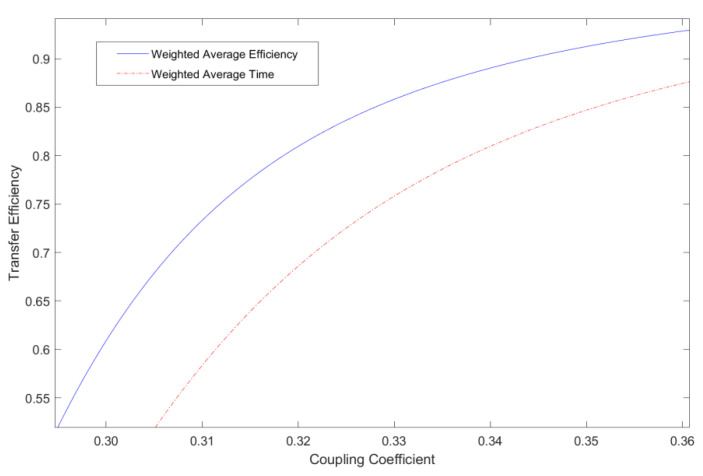
Efficiency curve corresponding to time-weighted coefficient method.

To further illustrate the feasibility of this method, this time, it will be compared with the time-weighted coefficient method. The time-weighted coefficient method is based on the different characteristics of the battery considered in different charging stages, and assigns weights to the efficiency values of different stages of battery constant current and constant voltage charging to obtain the optimal time-weighted average efficiency [21]. The efficiency curve obtained by using the time-weighted coefficient method after the coupling coefficient is shifted within [0.35, 0.31] and is shown in Figure 10.

According to Figure 9, on the premise that the influence of the coupling coefficient on the efficiency is mainly considered, compared with the time-weighted coefficient method, the offset weight coefficient method can obtain a higher transmission efficiency in the entire value range of the coupling coefficient. The efficiency improvement is more obvious when the offset distance is larger. Specifically, the simulation results show that when the coupling coefficient is 0.35 (corresponding to the small offset state), the efficiency of the method in this paper is increased by about 4%; when the coupling coefficient is 0.332 (corresponding to the medium offset state), the method in this paper achieves an increased efficiency of about 6.2%; when the coupling coefficient is 0.31 (corresponding to the large offset state), the transmission efficiency obtained by this method can still be maintained above 60%, while the efficiency obtained by the time-weighted coefficient method will be lower than 60%.

### 2.4. Analysis of the Fault Tolerance Capability of the Coupling Device

**Figure 11:**
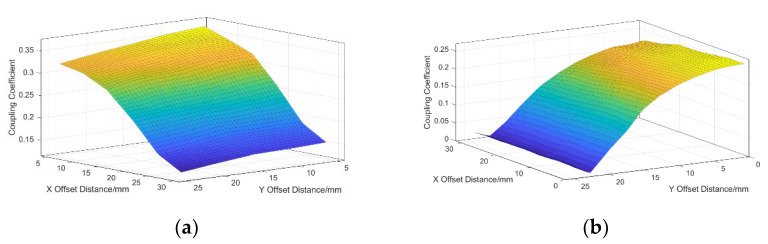
The change diagram of the coupling coefficient along X–Y bidirectional offset. (**a**) New orthogonal coupler; (**b**) bipolar device.

To test the anti-offset capability of the coupling device, the coupling coefficient changes of the new orthogonal coupling device and the orthogonal coupling device shown in Figure 3 are compared and analyzed when they are offset in the *X*-axis and *Y*-axis directions, and the simulation results are shown in Figure 11.

Figure 10 shows that, in the absence of offset, the coupling coefficient of the coupling coil designed this time is 27.8% higher than that of the traditional bipolar coil, and it is offset in the X direction or Y direction, respectively. The new orthogonal coupling structure has better anti-bias performance. When the *X*-axis is offset by 35 mm, the coupling coefficient between the coils is 28.5% higher than that of the bipolar type. When the *Y*-axis is offset by 20 mm, the coupling coefficient between the coils is 28.5%. Compared with the bipolar type, it has increased by 36.4%, which well-compensates for the problem that the coupling coefficient drops too much when the bipolar type is offset in the Y direction. When the *X*-axis direction is offset by 30 mm and the *Y*-axis direction is offset by 20 mm, the drone is in a large-scale offset state at this time, and the coupling coefficient of the new orthogonal coupling device is 66.7% higher than that of the bipolar device, which shows that the new orthogonal coupling device has the better anti-offset capability.

To further test the ability to resist multidimensional migration, the receiving coil is rotated based on migration along the *X*-axis, and the coupling coefficient changes of the new orthogonal coupling device and bipolar device are simulated and analyzed.

**Figure 12:**
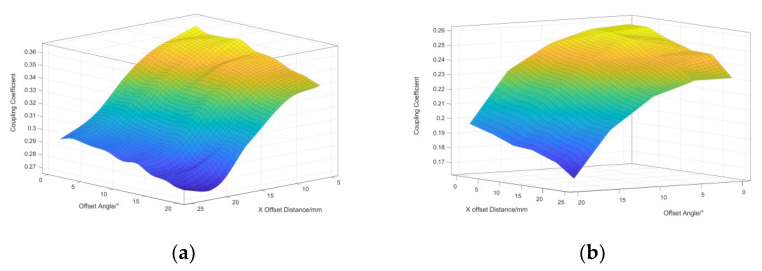

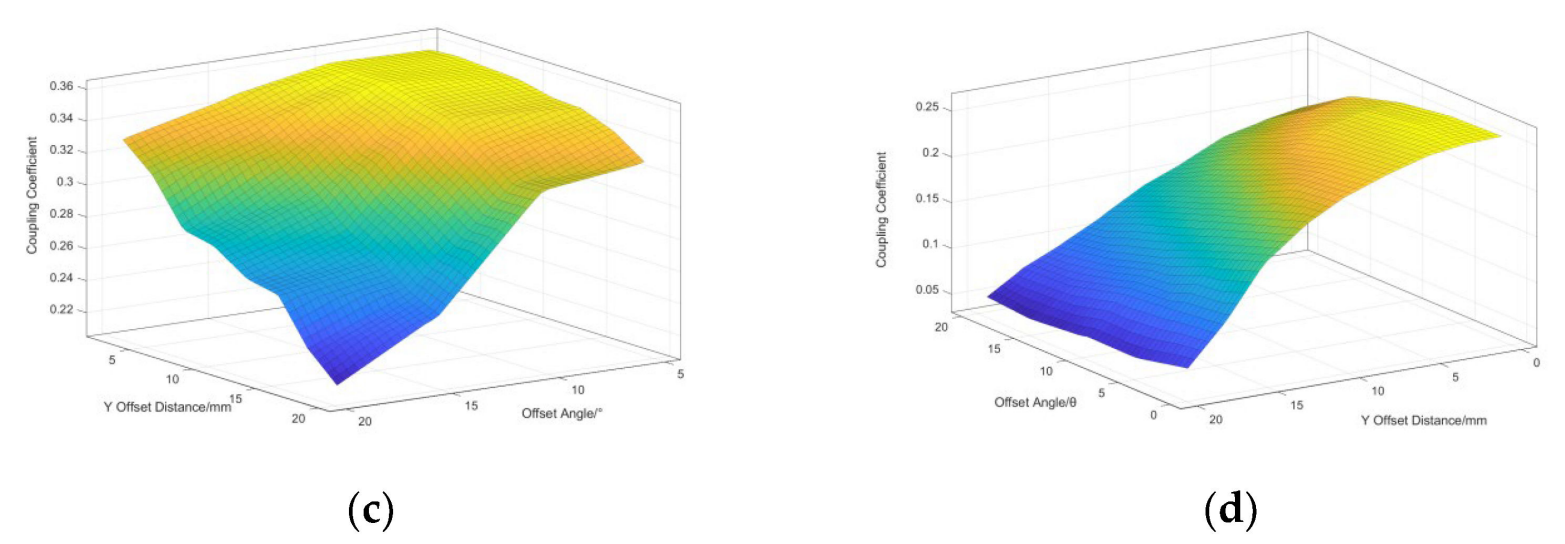
The change diagram of the coupling coefficient along angular offset. (**a**) New orthogonal coupler; (**b**) bipolar device; (**c**) new orthogonal coupler; (**d**) bipolar device.

As can be seen from Figure 12, the test results show that when the rotation angle is 20° and the deviation is 25 mm along the *X*-axis, the coupling coefficient of the new orthogonal coupling device increases by 0.11 compared with that of the bipolar device. When the rotation angle is 20° and the *Y*-axis is offset by 20 mm, the coupling coefficient of the new orthogonal coupling device is increased by 0.15 compared with that of the bipolar device. which indicates that the new orthogonal coupling device has better anti-deviation ability.

## 3. Design of Constant Current Control Strategy for UAV-WPT

### 3.1. Secondary Side Constant Current Control Strategy Based on Model Predictive Control

**Figure 13:**
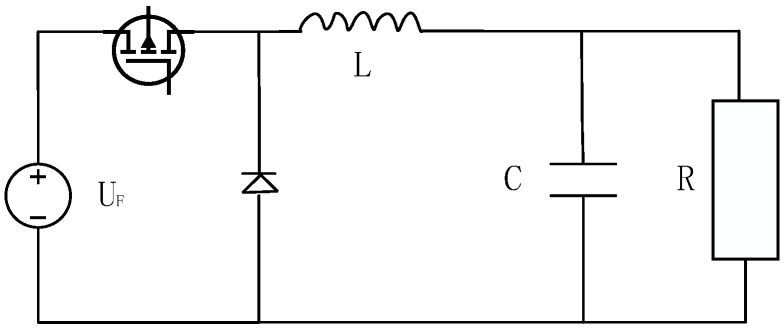
Buck circuit topology.

To realize the control of the wireless charging system for small UAVs, it is first necessary to establish a mathematical model of the control object. Since the input voltage is provided by the wireless charging side, the power input end of the buck circuit can be simplified as a power supply during the modeling process. The control of the buck circuit facilitates the control of the output current. The simplified model is shown in Figure 13. For this model, the state-space averaging method is used to describe the dynamic characteristics of the circuit. Based on ignoring the state transition time of the switch tube, i_L_, and V_c_ are used to represent the current flowing through the inductor and the voltage across the capacitor, respectively.

At $0\leq t\leq dT_{S}$, the switch tube is in the conduction state, the diode is in the cut-off state at this time, the inductor stores energy through the power supply terminal, and the load R is supplied with energy by the capacitor C. The system state equation at this time is:

(11) $$\left\{\begin{array}{l} L\frac{di_{L}(t)}{dt}=V_{F}-V_{C}-i_{L}R_{L}\\ C\frac{dV_{C}(t)}{dt}=i_{L}-\frac{V_{C}}{R} \end{array}\right.$$

At $dT_{S}\leq t\leq T_{S}$, the switch tube is in the off state, and the inductance supplies power to the load R and the capacitor C at this time. The state equation of the system at this time is:

(12) $$\left\{\begin{array}{l} L\frac{di_{C}(t)}{dt}=-i_{L}R_{L}-V_{C}\\ C\frac{dV_{C}(t)}{dt}=i_{L}-\frac{V_{C}}{R} \end{array}\right.$$

The state variable $x={\left[V_{C}(t),i_{L}(t)\right]}^{T}$, u=d(t) is selected at this time through Formulas (11) and (12) at the k switching cycle of the switching tube $T_{S}$, whose on and off state equations are shown in Formula (13):

(13) $$\left\{\begin{array}{l} \dot{x}=G_{1}x+H_{1}u,kT_{S}\leq t\leq (k+d)T_{S}\\ \dot{x}=G_{2}x+H_{2}u,(k+d)T_{S}\leq t\leq (k+1)T_{S} \end{array}\right.$$

where G_1_, H_1_, G_2_ and H_2_ are:

$$G_{1}=\left[\begin{array}{cc} -\frac{1}{L} & -\frac{R_{L}}{L}\\ -\frac{1}{CR} & \frac{1}{C} \end{array}\right],\;H_{1}=\left[\begin{array}{c} \frac{V_{F}}{L}\\ 0 \end{array}\right],\;G_{2}=\left[\begin{array}{cc} -\frac{1}{L} & -\frac{R_{L}}{L}\\ -\frac{1}{CR} & \frac{1}{C} \end{array}\right],\;H_{2}=\left[\begin{array}{c} 0\\ 0 \end{array}\right]$$

In the case of ensuring that the sampling period and the switching period do not change, the discrete-time state equation can be obtained by discretizing Formula (14):

(14) x(k+1)=Ax(k)+BD(k)

Assuming that the turn-on and turn-off times of the switch tube is D, the state space averaging method can be used to obtain:

(15) $$\left\{\begin{array}{l} x(k+1)=A_{d}x(k)+B_{d}D(k)\\ y=C_{d}x(k) \end{array}\right.$$

Among them, $A_{d}=1+AT$, $B_{d}=BT$, $C_{d}=[1/R,0]$.

### 3.2. Model Predictive Controller Design

According to the principle of the model predictive control algorithm [22], the augmented matrix is established based on Formula (16), as shown in Formula (17).

(16) $$\left[\begin{array}{l} \Delta X(k+1)\\ y(k+1) \end{array}\right]=\left[\begin{array}{cc} A_{d} & O_{N}\\ C_{d}A_{d} & 1 \end{array}\right]\left[\begin{array}{l} \Delta X(k)\\ y(k) \end{array}\right]+\left[\begin{array}{l} B_{d}\\ C_{d}B_{d} \end{array}\right]\Delta D(k)=A_{e}\left[\begin{array}{l} \Delta X(k)\\ y(k) \end{array}\right]+B_{e}\Delta D(k)$$

(17) $$y(k)=\left[\begin{array}{cc} O_{N} & 1 \end{array}\right]\left[\begin{array}{c} \Delta X(k)\\ y(k) \end{array}\right]=C_{e}\left[\begin{array}{c} \Delta X(k)\\ y(k) \end{array}\right]$$

where $O_{N}$ is a zero vector.

To enhance the controller’s ability to track system state changes and target physical quantities, as well as improve the robustness to system changes, it is possible to expand the dimension of the state vector by establishing an augmented matrix; and thus, the error of the model parameter drift is improved. Using the augmented matrix, the prediction equation of the system in the future N_P_ sampling moments can be extended to better reflect the changing trend of the system state. That is, the augmented matrix can expand the state vector of the system so that the controller can understand the state changes of the system more comprehensively and make more accurate control decisions. The prediction equation of the system in N_P_ sampling time in the future is:

(18) $$Y=F\left[\begin{array}{c} \Delta X(k)\\ y(k) \end{array}\right]+\Phi \Delta D(k)$$

In Formula (17),

$$Y=\left[\begin{array}{l} y(k+1|k)\\ y(k+2|k)\\ \ldots \\ y(k+N_{p}|k) \end{array}\right],\;\Delta D=\left[\begin{array}{l} \Delta D(k)\\ \Delta D(k+1)\\ \cdots \\ \Delta D(k+N_{C}-1) \end{array}\right],\;F=\left[\begin{array}{l} C_{e}A_{e}\\ C_{e}A_{e}{}^{2}\\ \cdots \\ C_{e}A_{e}{}^{N_{p}} \end{array}\right],\;\;\Phi =\left[\begin{array}{cccc} C_{e}B_{e} & 0 & \cdots & 0\\ C_{e}A_{e}B_{e} & C_{e}B_{e} & \cdots & 0\\ \vdots & \vdots & \vdots & 0\\ C_{e}A_{e}{}^{N_{p}-1}B_{e} & C_{e}A_{e}{}^{N_{p}-2}B_{e} & \cdots & C_{e}A_{e}{}^{N_{p}-N_{c}}B_{e} \end{array}\right].$$

N_C_ is the length of the control sequence, and N_P_ is the step size of the prediction time domain. Among them, the prediction time-domain step size directly affects the stability and robustness of the system. Generally, the stability of the control system can be enhanced by increasing the prediction time-domain step size, but too large a prediction time-domain step size will lead to rapid system failure performance deterioration, affecting the dynamic response speed of the system. In a general predictive control system, it is necessary to ensure that the length of the control sequence is greater than the predicted time domain step size. To ensure the stability and robustness of the system, the time domain step size N_P_ is set to 5, and the control sequence prediction step size N_C_ is 3.

Therefore, the cost function J can be established according to the above parameters, as shown in Formula (19):

(19) $$J={\left(R_{S}-Y\right)}^{T}Q(R_{S}-Y)+\Delta D^{T}R\Delta D$$

Formula (18), when applied to a quadratic programming problem, can be expressed as:

(20) $$J=\Delta D^{T}F_{N}+\Delta D^{T}E\Delta D$$

(21) $$F_{N}=2\Phi QF\left[X(k)-R_{r}(k)\right]$$

(22) $$E=\Phi^{T}Q\Phi +R$$

In the formula, R_r_(k) = [0, …,0, ref(k)]^T^ is a column vector with the same dimension as X(k); ref(k) represents the target reference value at time k; Q and R, respectively, are a weight matrix composed of weight constants $q_{w}$ and $r_{w}$. Increased values in the weight matrices Q and R. $q_{w}$ can speed up the dynamic response of the system but lead to a decrease in system stability, and vice versa. In this design, the weight constant $q_{w}$ = 1 and $r_{w}$ = 10^−5^ is chosen to achieve higher control precision.

By solving a quadratic programming problem,

$$\underset{\Delta D}{Min}J=\Delta D^{T}F_{N}+\Delta D^{T}E\Delta D\;s.t.\;\;0.1\leq \Delta D\leq 0.9$$

**Figure 14:**
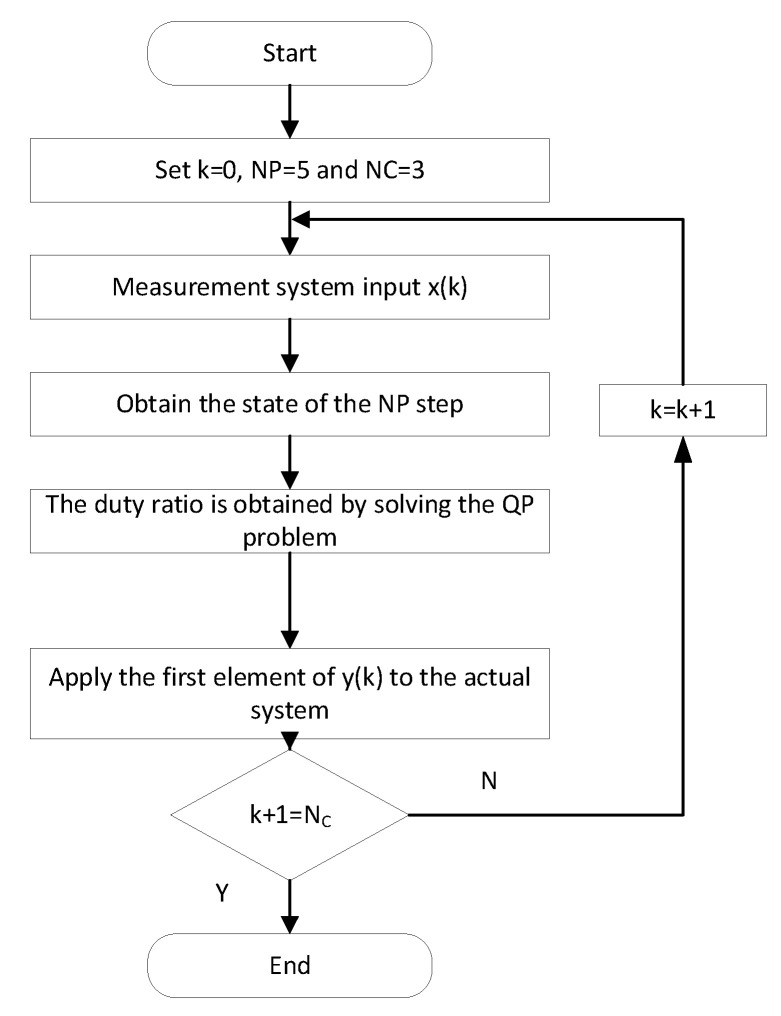
Flow chart of model prediction control.

The optimal duty ratio control signal at the current moment can be obtained ΔD. To cope with the interference brought by various external environments and device parameter changes to the system, the process will be executed in each sampling period in a rolling optimization manner. If the rated power output needs to be achieved, it can be achieved by changing the current reference value. The constant current control method program based on model prediction is written on the MATLAB platform, and the algorithm flow is shown in Figure 14.

### 3.3. Simulation Verification

To verify the effectiveness of the secondary side power control combined with the model prediction control, the LCC-S topology simulation model was built in the MATLAB/Simulink environment. The values of the WPT system for small UAVs are as mentioned above, whereby buck circuit parameters take the filter inductance 22 μH and capacitance 5.2 nF. In the simulation, the reference current, mutual inductance, and load values are set as periodic jumps to simulate the impact of transmission distance changes, load mutations, and coil offsets on the system output in actual applications.

**Figure 15:**
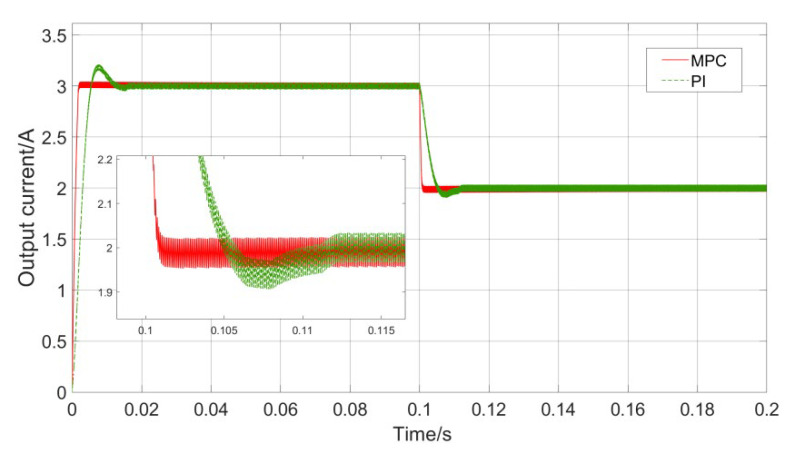
Reference current step change control comparison.

The response results of the model predictive controller and PI controller to the step reference signal are shown in Figure 15.

The initial reference value of the load current is set to 3 A, and a step change occurs at 0.1 s. It can be seen that both sets of controllers can accurately track the step change of the reference value, and there is no significant difference in tracking accuracy, but there are significant differences in tracking speed and overshoot. When the reference voltage jumps from 3 A to 2 A, the MPC controller essentially has no overshoot, and it only takes 0.6 ms to complete the reference value tracking; the PI controller has a significant overshoot, and the response speed is still slower than MPC, and the excessive overshoot has the potential risk of affecting the safety of system components. It can be seen that the MPC control has obvious advantages over the PI control in terms of the speed of tracking the reference value, and it is more in line with the needs of the small UAV-WPT system for fast response.

**Figure 16:**
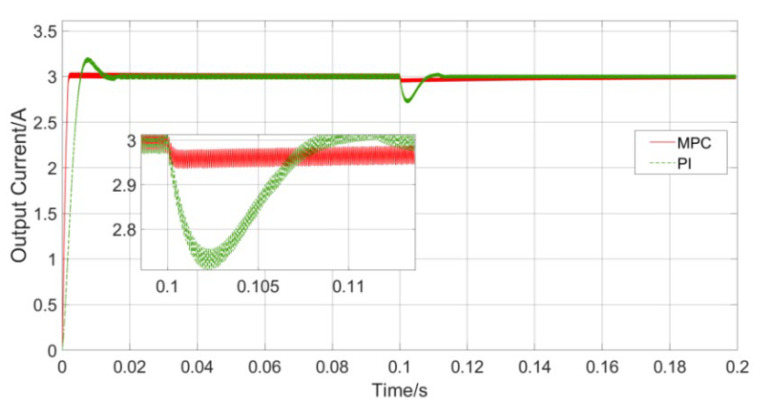
Mutual inductance change control comparison.

In addition to fast response, the controller also needs to deal with the impact of dynamic disturbances in the mutual inductance coefficient and load value in the small UAV-WPT system. The response waveforms of the MPC and PI controllers when the mutual inductance jumps are shown in Figure 16.

When jumps occur from 5 μH to 7 μH at 0.1 s, the input voltage amplitude will change abruptly. This process simulates the change of mutual inductance when the charging area is switched and the coil is offset in the small UAV-WPT system, and the controller needs to keep the load current as stable as possible under this condition. It can be seen from Figure 14 that the PI controller needs a certain adjustment time to stabilize the load current, but there is an obvious overshoot in the adjustment process. Therefore, the load current fluctuates obviously during charging under the action of the PI controller, while the fluctuation of the load current under the control of the MPC is always less than 0.5%. It can be seen that, compared with the PI controller, the MPC controller is more robust to changes in mutual inductance.

**Figure 17:**
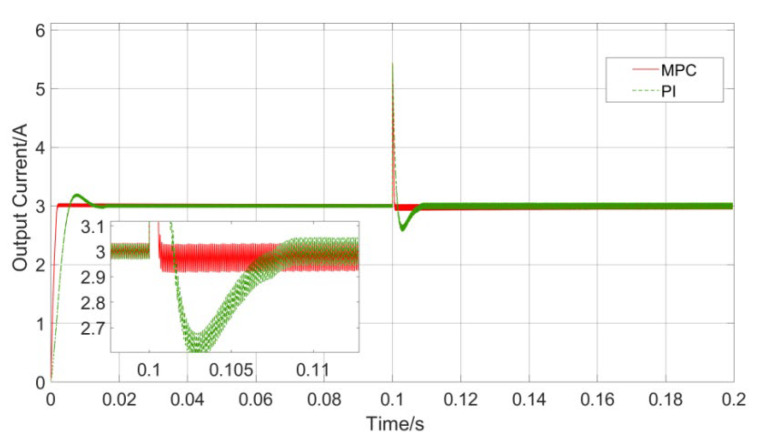
Load mutation control comparison.

Figure 17 shows the response waveforms of the MPC and PI controllers when the load jumps.

The load resistance value jumps from 20 Ω to 10 Ω at 0.1 s. It can be seen that both controllers produce obvious current spikes at the moment of the jump in the load resistance value. This is because the reference current value is set to 3 A before the jump, so that when the first jump occurs, the current change at both ends of the load. The voltage is still 20 V, so the current will change instantaneously, which is the reason for the current peak. During load switching, the regulation time of the MPC controller is 1.0 ms, while that of the PI controller is 3.9 ms, respectively. Therefore, when the load resistance changes, MPC control has obvious advantages in response speed compared with PI control. Combining Figure 15 and Figure 16, it can be seen that MPC has a strong robustness to the dynamic changes of mutual inductance and load, and can better meet the robustness requirements of the small UAV-WPT system.

## 4. Experiments and Result Analysis

**Figure 18:**
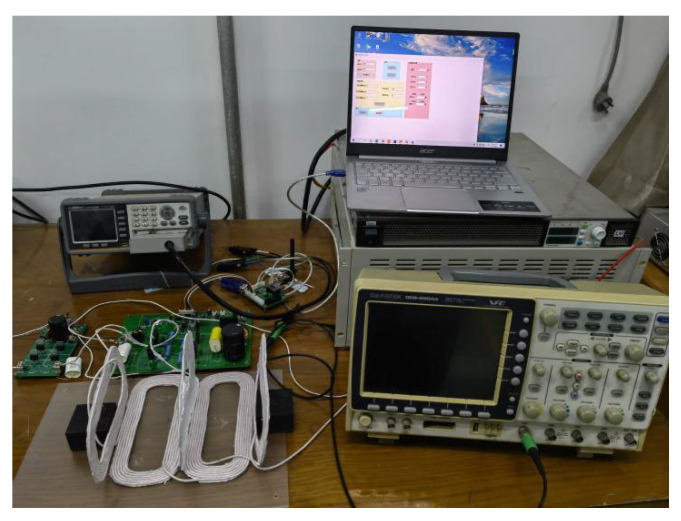
Wireless charging system experimental platform.

In this experiment, the coil of the coupling device is wound with a high-frequency Litz wire with a diameter of 3.5 mm. The operating frequency of the system is set at 85 kHz. After measuring the actual weight of the receiving coil after winding, the weight of the coil is 47 g, accounting for only about 9% of the weight of the UAV. It can be seen that the coupling mechanism has little effect on the weight of the UAV and can be conveniently installed on the drone. The system is composed of a DC power supply, a high-frequency inverter, a rectifier filter unit, and an electronic load, as shown in Figure 18.

The circuit parameters are shown in Table 4.

To test the anti-offset capability of the wound coupling device, when the phase shift angle of the inverter driving signal of the system is fixed at 30°, the load resistance is set to 5 Ω, and the system is kept in a positive condition. The input DC voltage is set to 24 V. The output current is 4 A, and the system working efficiency and output current change of the coupling mechanism in single-dimensional and multi-dimensional cases are tested.

### 4.1. Constant Current Capability Experiment

**Figure 19:**
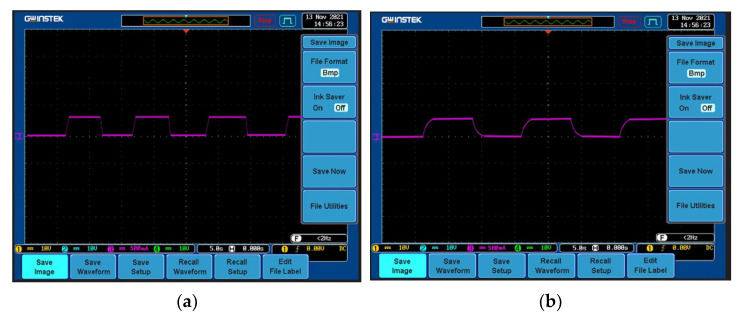
Reference current changes suddenly. (**a**) MPC controller; (**b**) PI controller.

To experiment with the constant current capability of the system, the reference current value is set to 3 A. First, the two controllers are verified experimentally by changing the reference current value to 3.4 A. The MPC and PI controllers control the reference current. The tracking results are shown in Figure 19a and Figure 19b, respectively. The PI controller needs about 1.3 s to complete the tracking, and when the reference value returns to 3A, the adjustment time of the PI controller is about 2.1 s. However, the MPC controller only needs about 0.7 s to complete the reference value tracking in the above two jumping processes. It can be seen that although both methods can achieve control accuracy and no overshoot, the tracking of the current value, the adjustment time required by the MPC controller, is significantly shorter than that of the PI controller, and the response speed is faster.

**Figure 20:**
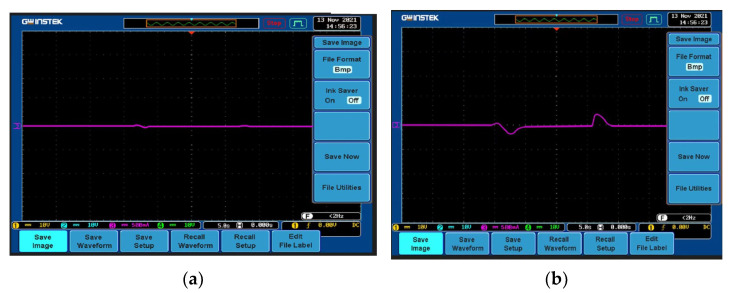
Mutual inductance changes suddenly. (**a**) MPC controller; (**b**) PI controller.

To realize the sudden change of mutual inductance, this phenomenon is simulated by moving the coil at the transmitting end in this experiment. The tracking results of reference current by MPC controller and PI controller in the case of mutual inductance mutation are shown in Figure 20a and Figure 20b, respectively. It can be seen from the experimental results that both the MPC controller and the PI controller can quickly and accurately track the set reference current value of 3A during the coil movement, but the average adjustment time of the PI controller is about 3 s, while the MPC controller can almost adjust the whole process with an average adjustment time of 1.3 s. However, compared with the PI controller, the MPC controller is more stable in the process of adjusting the output current, which proves that the MPC controller has stronger robustness.

**Figure 21:**
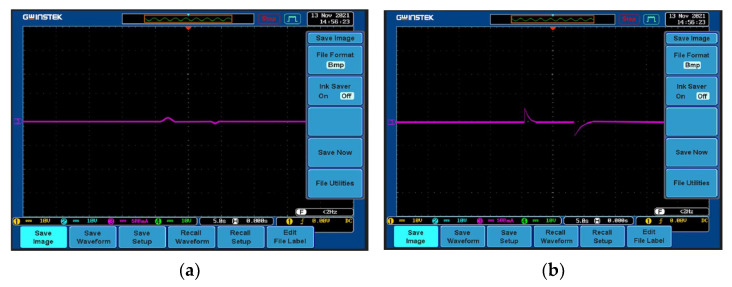
Load resistance changes suddenly. (**a**) MPC controller; (**b**) PI controller.

To realize the sudden change in the load, the method of switching the load resistance value is implemented in this experiment. In the experiment, the load is first reduced from 10 Ω to 5 Ω and then increased to 10 Ω. In this case, the tracking results of the reference current 3A by the MPC and PI controllers are shown in Figure 21a and Figure 21b, respectively. Specifically, the average adjustment time of the PI controller is about 2.1 s, while the average adjustment time of the MPC controller is only 1.3 s. The experimental results show that compared with the PI controller, the output current of the MPC controller is less than that of the PI controller in the case of sudden load changes.

### 4.2. System Work Efficiency Experiment

**Figure 22:**
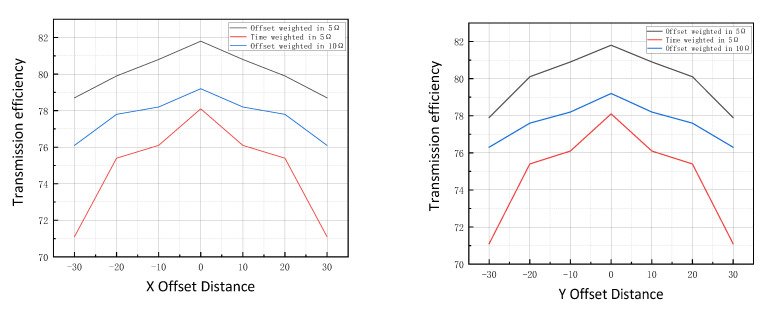
Transmit efficiency under offset.

To further verify the effect of the proposed efficiency strategy, when the reference output current is kept constant, the system coupling coil is offset on the X and Y axes and the load is changed to ensure that the system output power is not lower than 50 W. The transmission efficiency of the two systems before and after optimization is compared as shown in Figure 22.

It can be seen from Figure 22 that when the coupling coefficient of the system decreases sharply with the increase in load resistance, the influence of the load change on the efficiency is more obvious in this experiment. By designing the coupling parameters using the offset weight method, the coupling coefficient between coils can be improved during the offset, and thus also the magnetic induction intensity of emitting coils in space, so as to improve the output efficiency of the system.

## 5. Conclusions

In this paper, the equivalent circuit model of the LCC-S topology is first established. Based on the new orthogonal coupling device, according to the influence of the coupling coefficient change on the transmission efficiency caused by the coil offset, a probability model based on normal distribution is proposed. The offset weight method is used to complete the design of the system resonance parameters and improve the anti-offset capability of the UAV-WPT device; secondly, a constant current control strategy based on model prediction is proposed, and the duty cycle of the buck circuit is controlled through the model prediction algorithm, so that the system can still maintain a constant current output when the coupling coefficient, the load, and the reference current change, making the system more practical. The experiment results show that the coupling device designed this time can improve the anti-offset capability of the system, and the proposed control strategy can improve the system parameter efficiency based on realizing constant current output.

However, only the load was used instead of the battery in the experiment this time, which is the limitation of the current research. On the basis of this study, future research can study the control method for constant current and constant voltage charging to meet the actual needs of wireless charging for drones.

## Figures and Tables

**Table 1:** Dimensions of the new orthogonal coupling device.

Parameter	Value	Parameter	Value
Transmitting coil length	140 mm	Receive coil length	120 mm
Total width of transmitting coil	90 mm	Receive coil width	5 mm
Transmitter coil diameter	20 mm	Receiver coil height	45 mm

**Table 2:** The corresponding weights under different offsets.

X and Y Offset Distance/mm	Probability	Weight Size	X and Y Offset Distance/mm
[−10, 10]	57.4%	0.57	[−10, 10]
[−20, −10] U [10, 20]	27.9%	0.28	[−20, −10] U [10, 20]
[−30, −20] U [20, 30]	14.4%	0.15	[−30, −20] U [20, 30]

**Table 3:** Parameter result table.

	$L_{f}/\mu H$	$C_{f}/nF$	$C_{1}/nF$	$C_{2}/nF$
Offset weight method/mm	7.8	446	86	70
Time-weighted method/s	11.1	320	90	70

**Table 4:** Circuit parameters.

Parameter	Symbol	Value
Inductance of coil	L_1_, L_2_	80 μH, 15 μH
Compensation inductance	L_f_	8 μH
Resonance capacitance	C_1_, C_2_	90 nF, 70 nF
Compensation capacitance	C_f_	450 nF
Load resistance	R_L_	5 Ω

## Data Availability

Not applicable.
